# Active physical human-exoskeleton interaction based on motion intention adaptive recognition and synchronous tracking

**DOI:** 10.3389/fnbot.2026.1779125

**Published:** 2026-05-20

**Authors:** Weiguo Shi, Weiqun Wang, Jiaxing Wang, Shengda Liu, Xu Liang, Tianyu Lin, Kexin Xiang, Zeng-Guang Hou

**Affiliations:** 1 School of Artificial Intelligence, University of Chinese Academy of Sciences, Beijing, China; 2 State Key Laboratory of Multimodal Artificial Intelligence Systems, Institute of Automation, Chinese Academy of Sciences, Beijing, China; 3 School of Automation and Intelligence, Beijing Jiaotong University, Beijing, China

**Keywords:** active rehabilitation, gait training, lower limb exoskeleton, sEMG based motion intention recognition, synchronous tracking

## Abstract

Active physical human–exoskeleton interaction has been widely studied. However, the challenges of human motion intention recognition and synchronous tracking have not been well-addressed. In this article, a motion intention recognition method based on biophysical information fusion and adaptive learning was proposed to overcome the limitations of existing approaches. First, a lower-limb joint angle prediction model was developed by integrating surface electromyography (sEMG), historical joint angles and centers of gravity. The convolutional neural network, Mamba network, and multilayer perceptron network were used respectively for feature extraction, information fusion, and joint angle prediction. Second, an online adaptive method for the angle prediction model was designed based on a style transfer mapping technique to address the issue of recognition accuracy decline. In this method, the new sEMG features were mapped into the initial feature space, by which the prediction model can maintain the predictive performance during long-term implementation. Furthermore, a real-time control method for the exoskeleton synchronous tracking was given based on the predicted angles. Finally, the feasibility of the proposed methods was validated through the offline and online experiments.

## 1 Introduction

Active physical human-robot interaction is very important for the human-robot coupled systems, including exoskeletons, rehabilitation robots and intelligent prostheses (Luo et al., 2024). In order to implement the active interaction in these systems, human motion intentions should first be recognized; however, it has not been well-addressed (Wang et al., 2023; Liang et al., 2022). Presently, most methods for recognizing human motion intentions were designed using physiological or physical signals (Liang et al., 2024). The physiological signals, such as surface electromyography (sEMG) (Wang et al., 2024) and electroencephalography (EEG) (Wang et al., 2021; Lorach et al., 2023), have been widely applied for recognition of human motion intentions, and sEMG based methods can outperform others in reliability and decoding accuracy.

sEMG signals have been frequently used to classify discrete motion patterns or to predict continuous motion quantities in the literature (Li et al., 2020b). Physical gestures were mainly focused on for classification of discrete motion patterns (Li et al., 2019; Zhu et al., 2022; Zeng et al., 2022). The main goal of the continuous motion estimation is to establish the relationships between sEMG data and joint dynamic parameters (Sitole and Sup, 2023; Tian et al., 2024), such as joint torques, velocities and angles.

Although significant progress has been made in sEMG based motion intention recognition, their practical application is still limited due to the prediction accuracy degradation, which frequently appears during online implementment of the pre-trained recognition model (Wei et al., 2024; Kyranou et al., 2025; Spanias et al., 2015). This degradation is mainly caused by the inherent non-stationary property of sEMG signals, which is related to various factors, such as muscle fatigue (Cifrek et al., 2009), electrode displacement (Teh and Hargrove, 2020), and differences in body posture (Xu and Xiong, 2021), etc. Several approaches have been proposed to improve the robustness and adaptability of sEMG based motion intention recognition methods. The first approach involves the application of adaptive learning algorithms, by which model parameters can be updated in real-time (Ameri et al., 2020). The second approach involves integrating sEMG signals with other signals, such as inertial measurement units (IMUs) (Delgado et al., 2021). Transfer learning techniques were also applied to transfer knowledge from previous subjects or tasks, by which extensive subject-specific training can be reduced and cross-subject generalization can be enhanced (Zhang et al., 2022; Bao et al., 2021; Pan et al., 2025). Moreover, advanced signal processing methods, such as wavelet transforms and time-frequency analysis, have been used to extract features less sensitive to muscle fatigue and electrode shifts (Rani et al., 2024; Su et al., 2026). However, two challenges have not been well-addressed in most existing methods. First, many adaptive learning and transfer learning techniques are specifically designed for the particular models, such as deep neural networks or support vector machines, due to which the general applicability across different models is limited. Second, effective feature extraction and data fusion methods for different signals are not sufficiently developed, by which the full exploitation of the complementary characteristics among heterogeneous multimodal data is limited.

By considering the limitations of current methods, a solution was proposed by integrating a lower-limb joint angle prediction model, an adaptive method, and a control framework for the exoskeleton in this study.

First, a lower-limb joint angle prediction method based on multimodal data fusion and a hybrid neural network was proposed. Specifically, sEMG signals, centers of gravity (CoGs), and joint angles were collected synchronously as the inputs of the joint prediction model. To fully exploit information from the heterogeneous multimodal data, one-dimensional convolutional neural networks (1D-CNNs) were employed for feature extraction, and a Mamba network was utilized for information fusion. Subsequently, a multilayer perceptron (MLP) network was constructed as the mapping module, and the outputs of the MLP were the predicted joint angles.

Second, an adaptive method for prediction of joint angles was developed based on real-time update of a transfer mapping matrix. Source and target domain datasets were constructed from offline and online sEMG samples, respectively. The principal component analysis (PCA) and style transfer mapping (STM) methods were applied to establish the transfer mapping matrix, which was iteratively updated based on the prediction errors and the source domain dataset.

Third, a two-layer collaborative control framework was designed for the exoskeleton. At the high-level layer, the subject's lower-limb joint angles were predicted using the proposed method and taken as the control input of the exoskeleton. At the low-level layer, a non-linear constraint and the bilateral filtering method were employed to improve safety of the human-exoskeleton collaboration. Moreover, a position-velocity hybrid strategy based on error feedback was used to reduce cumulative errors.

Finally, extensive experiments were conducted to validate the feasibility of the proposed methods. It was demonstrated by the results of ablation experiment that the prediction accuracies were significantly improved by fusion of joint angles and CoG data. At the same time, the proposed hybrid neural network outperformed existing approaches in the prediction accuracies. The pseudo-online and online experiments demonstrated also that the joint angle prediction errors were effectively reduced by the proposed online adaptive method. Additionally, the human-exoskeleton collaborative experiments were performed as well. The results indicated that the subject's movement could be synchronously tracked by the exoskeleton motion.

The remainder of this article is organized as follows. In Section 2, the joint angle prediction method based on multimodal data fusion and the hybrid neural network is presented. In Section 3, the online adaptive approach for the prediction model is introduced. In Section 4, the hierarchical collaborative control framework is proposed for the human-exoskeleton interaction. The experiments and results are presented in Section 5, and conclusions and future work are presented in Section 6.

## 2 Model design for recognition of human motion intentions

**Figure 1:**
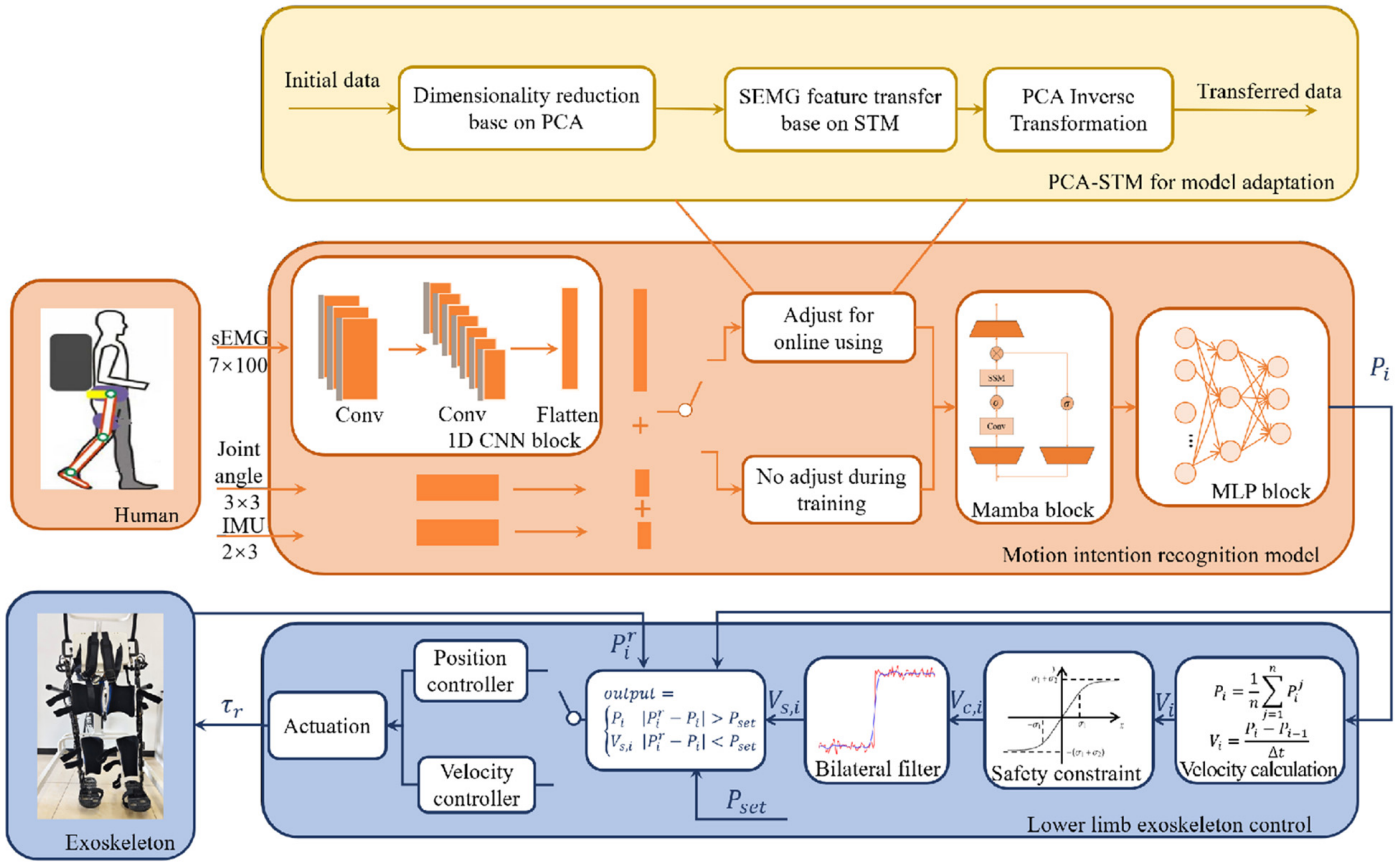
Intention driven human-exoskeleton interaction control framework. It consists of two parts, namely the high-level intention adaptive recognition based on the proposed PCA-STM method and low-level position and velocity control. In real-time operation, sEMG, historical joint angles and IMU data were used to predict the next joint angles and then the predicted joint angles was applied for the exoskeleton synchronous tracking.

The joint angle prediction model was designed based on multimodal data, including sEMG signals, CoG data and joint angles. A hybrid neural network was designed, where the 1D-CNN was first employed to extract features from the multimodal input data. The extracted features were then effectively fused through the Mamba network. Finally, the outputs of Mamba network were fed into the MLP network to predict the joint angles of the lower limbs, as illustrated in Figure 1.

### 2.1 Feature extraction

**Figure 2:**
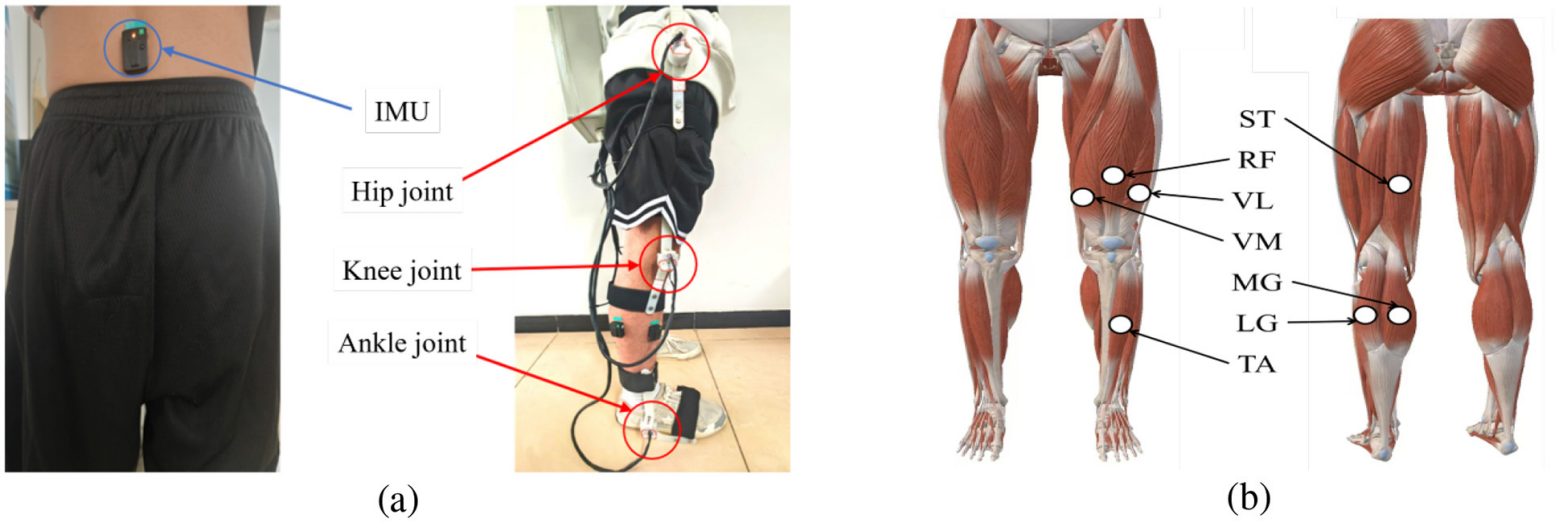
The sensor placement and selected muscles. **(a)** The placement of sensors on the human body. The red circles show the sEMG joint angle sensors, the blue circle shows the IMU sensor. **(b)** The selected muscles in this study.

The CNNs have been widely applied in processing time series, which can effectively capture spatial and temporal features (Li et al., 2022). In this study, the 1D-CNN was employed to extract features from sEMG signals, joint angles and CoG data. The multimodal data were acquired through sensors strategically placed on the lower limbs and trunk to capture both muscular activity and kinematic information, as shown in Figure 2.

The sEMG signals were collected from seven muscles of the lower limbs, including the rectus femoris (RF), vastus medialis (VM), vastus lateralis (VL), semitendinosus (ST), medial gastrocnemius (MG), lateral gastrocnemius (LG) and tibialis anterior (TA). The sEMG signals were sampled an 2,000 Hz by a wireless sEMG system (Delsys, Trigno^TM^, Natick, MA, United States). In the data preprocessing, the continuous sEMG signals were segmented using the sliding window approach, where each window contained 100 data points, and each segment was treated as an individual sample. The 1D-CNN was then employed to automatically extract features from the segmented sEMG signals. The network architecture consisted of two sequential 1D-CNN layers, and the detailed configuration of each layer was summarized in Table 1. After passing through the convolutional layers, the resulting features were flattened into a 294-dimensional feature vector, providing a representation for the subsequent model processing.

**Table 1:** Architecture of the proposed model.

Module	Layer type	Input→output	Hyperparameters
Feature extraction	sEMG input	7 × 100	–
	Conv1d	7 × 100 → 49 × 20	Kernel Size = 5, Stride = 5
	Conv1d	49 × 20 → 49 × 6	Kernel Size = 5, Stride = 3
	Flatten	49 × 6 → 294	–
	Angle + CoG	5-dim	–
Feature fusion	Concatenation	294+5 → 299	–
Prediction output	Mamba × 2 Linear + ReLU Linear + ReLU Linear	(299, 1) → (299, 1) 299 → 64 32 → 32 32 → 3	*D*_*state*_ = 16, *D*_*conv*_ = 4 – – –

The CoG data were recorded from an IMU sensor mounted on the sacrum, capturing vertical (up-down) and mediolateral (left-right) movements of the body's center of gravity. The signals were sampled at 60 Hz, and each sample contained three data points to align with the sEMG features for subsequent multimodal data processing.

Joint angles were measured from the hip, knee, and ankle joints using a custom-built sensor system. The signals were sampled at 60 Hz, and each sample contained three data points, by which the joint angles were aligned with the sEMG segments.

The multimodal data were aligned based on timestamps. Each sEMG window (100 points, 50 ms) was synchronized with the kinematic data by selecting three CoG and joint angle samples within the corresponding time interval. Average filtering was applied to the three kinematic data points to generate a single representative value for each variable, by which reducing measurement noise can be reduced. A common software trigger signal was conducted to ensure temporal consistency across modalities.

### 2.2 Multimodal data feature fusion

The features extracted from sEMG signals, CoG data and joint angles were first concatenated into a unified vector. To further explore the deeper multimodal information, the vector was then fed to the Mamba network. The Mamba network was selected as the fusion model for the following reasons. First, a selective state-space mechanism is employed by the Mamba network to dynamically adjust state transitions based on input content, by which both short-term and long-term temporal patterns in multimodal information can be effectively captured. Second, the computational complexity of Mamba network is lower than that of the transformer based sequence models, which is critical for real-time exoskeleton control.

Mamba network is a sequence model based on a structured state-space framework (Gu and Dao, 2023; Dao and Gu, 2024), of which the hidden state is updated through the following equations:


$$\begin{array}{l} g_{t}=\text{H}g_{t-1}+\text{F}u_{t}, \end{array}$$ (1)

$$\begin{array}{l} y_{t}=\text{K}g_{t}, \end{array}$$ (2)

where **g**_*t*_ is the hidden state vector. **u**_*t*_ is the input vector. **H** represents the state transition matrix. **F** and **K** denote the mappings from input to state and from state to output, respectively. In this study, **u**_*t*_ represents the multimodal data features. The matrix **H** governs the state transition, effectively modeling the long-range temporal dependencies in locomotion.

### 2.3 Angle prediction

The MLP network, as a widely used architecture in artificial neural networks, consists of multiple fully connected layers with non-linear activation functions. Capable of learning complex mappings and capturing high-dimensional relationships within the integrated feature space, the MLP network was employed to predict joint angles based on the fused multimodal data features.

The network structure used in this study consisted of two hidden layers, with 64 neurons in the first hidden layer and 32 neurons in the second. Each hidden layer was followed by the Relu activation function. Moreover, a fully connected layer was applied to produce the outputs, which consisted of the angles of the hip, knee, and ankle joints.

## 3 Online adaptive method

Generally, recognition methods based on data-driven models are highly sensitive to the quality and the distribution between training data and online data. In real time, the amplitude and frequency of sEMG signals are easily affected by factors such as sensor displacement, muscle fatigue, and individual variability. Consequently, a distributional shift of the sEMG features between the training and online data can be caused and the prediction accuracy can be degraded. To overcome this limitation, a STM method has been proposed, by which the feature space can be adapted in real time and the distributions can be aligned.

### 3.1 Style transfer mapping

STM method is an effective transfer learning method, which has been successfully applied in various pattern recognition tasks and has achieved satisfied performance. The definitions of STM methods are different, but the fundamental ideas are similar (Li et al., 2020a). In this study, the STM method is defined by an affine transformation, by which the target domain can be transferred to the source domain. The affine transformation used can be described by:


$$\begin{array}{l} d_{i}=\text{A}o_{i}+b, \end{array}$$ (3)

where **d**_*i*_ denotes a sample in the target domain dataset **T** and is named the destination, **o**_*i*_ denotes a sample in the source domain dataset **S** and is named the origin. The **A** and **b** are transformation matrix and transformation bias, respectively. The **A** and **b** can be optimized by the following least squares function:


$$\begin{array}{l} \underset{A\in R^{m\times m},b\in R^{m}}{\min}\overset{n}{\underset{i=1}{\sum }}||Ao_{i}+b-d_{i}{||}_{2}^{2}+\beta ||A-I{||}_{F}^{2}+\gamma ||b{||}_{2}^{2}, \end{array}$$ (4)

where $||\cdot {||}_{F}^{2}$ is the Frobenius norm of matrix and ||·||^2^ is the L2 norm of vector. *n* is the number of sEMG features. The strength of regularization for the transformation matrix and the bias term are controlled by β and γ, respectively. Considering the data scaling factor, the parameters can be given as:


$$\begin{array}{l} \beta =\tilde{\beta }\frac{1}{\epsilon }\text{Tr}\left(o_{i}o_{i}^{T}\right),\gamma =n\tilde{\gamma }, \end{array}$$ (5)

where *Tr*(·) is the trace of the matrix, ϵ represents the data dimensionality. $\tilde{\beta }$ and $\tilde{\gamma }$ can be selected between 1 and 3. It can be seen that Equation 4 is a convex quadratic programming problem. Thus, a closed-form solution is obtained and can be described by:


$$\begin{array}{l} A=QM^{-1},b=\frac{1}{\hat{f}}\left(\hat{d}-A\hat{o}\right), \end{array}$$ (6)

where


$$\begin{array}{l} Q=\overset{n}{\underset{i=1}{\sum }}d_{i}o_{i}^{T}-\frac{1}{\hat{f}}\hat{d}{\hat{o}}^{T}+\beta I, \end{array}$$ (7)

$$\begin{array}{l} M=\overset{n}{\underset{i=1}{\sum }}o_{i}o_{i}^{T}-\frac{1}{\hat{f}}\hat{o}{\hat{o}}^{T}+\beta I, \end{array}$$ (8)

$$\begin{array}{l} \hat{o}=\overset{n}{\underset{i=1}{\sum }}o_{i},\hat{d}=\overset{n}{\underset{i=1}{\sum }}d_{i},\hat{f}=n+\gamma . \end{array}$$ (9)

It should be noted that the matrix inversion is calculated in the equation. Considering the fact that **M** is symmetric and generally positive definite, it can be calculated effectively.

### 3.2 Mapping origin and destination

It can be seen that the solution of Equation 4 is unique when the mapping destination and mapping origin are given. In this study, the mapping origin is the data in *A*^**T**^. In other words, the transfer learning method is determined by the definitions of the mapping destination in *A*^*S*^.

In previous research (Zhang and Liu, 2011), the STM method was generally used to solve classification problems and the destination was generally defined as the transferred features. In this study, the relationship between the physiological information extracted from sEMG signals and the kinematic information represented by the joint angle was considered. The movement of joints is the result of the contraction of muscles. The destination of sEMG data was defined based on the kinematic state most similar to those of source domain and target domain datasets. To search for the most similar kinematic state, the joint angular velocity and angle were considered simultaneously. Taking into account the direction of joint movement, two types of angular velocity were represented by


$$\begin{array}{l} v_{1,i}=p_{i}-p_{i-1},v_{2,i}=p_{i+1}-p_{i}, \end{array}$$ (10)

where **p**_*i*_ is the *i*th joint angles vector from the source domain. The representative kinematic information can then be defined as **K**_*i*_ = [**p**_*i*_, **v**_1, *i*_, **v**_2, *i*_]. For the source data **K**_*i*_ and the target data **K**_*j*_, two rules were considered in the search process. One rule was that the sign of **K**_*i*_ and the sign of **K**_*j*_ should be the same. The other was that the distance between **K**_*i*_ and **K**_*j*_ should be as small as possible, which was defined by Mahalanobis distance (De Maesschalck et al., 2000).

### 3.3 PCA-STM method

In the hybrid neural networks, the extracted features of sEMG signals were 294 in dimension. Considering the computational complexity and the efficiency, the PCA method was employed to further improve the execution efficiency of the STM method.

First, the original feature vector **X** in **A**^*S*^ was processed into a lower-dimensional vector **X**′ using the PCA method. Second, the reduced-dimensional vector **X**′ was mapped to the corresponding feature **Y**′ using the STM algorithm, as described in Equation 3. Third, the feature *Y*′ was transformed back into the original feature space to generate the full-dimensional mapping destination *Y*. The transfer learning method designed for online calibration can be summarized in Algorithm 1.

Algorithm 1 PCA-STM algorithm for online feature adaptation 
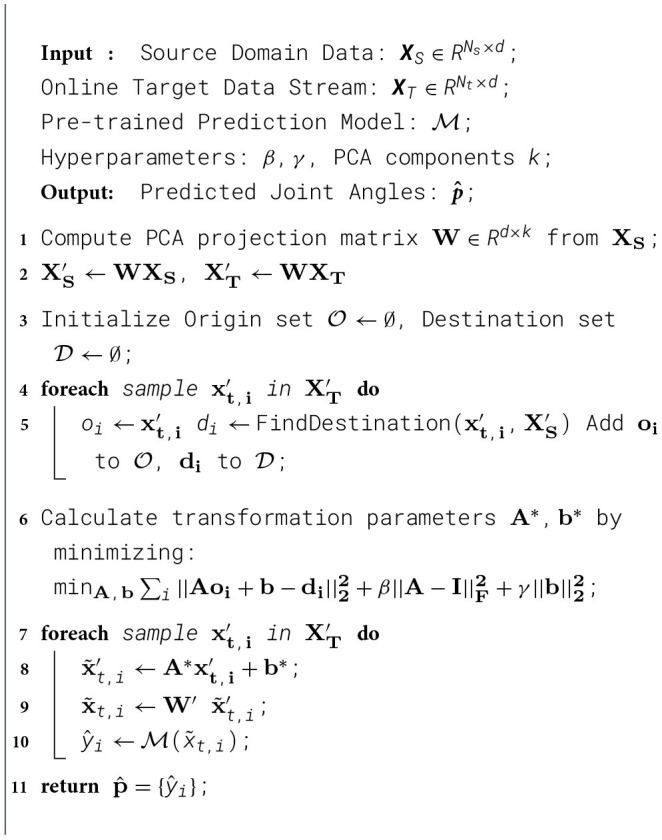

Whether to update the parameters **A** and **b** of the PCA-STM is another crucial aspect of the online experiment. In this study, the mean absolute percentage error (MAPE) was used for the update of the recognition model, which is defined by:


$$\begin{array}{l} \text{MAPE}=\frac{100\%}{n}\overset{n}{\underset{i=1}{\sum }}|\frac{{ŷ}_{i}-y_{i}}{y_{i}}|. \end{array}$$ (11)

Specifically, the performance of the model is continuously monitored, and when the MAPE exceeds the predefined threshold, the parameters were updated using the data from 30 s prior to the detected change.

## 4 Exoskeleton control framework

Based on the predicted joint angles, a real-time exoskeleton control strategy was subsequently developed for active physical human-exoskeleton interaction, as shown in Figure 1. The control framework was designed to address two practical challenges in intention-driven exoskeleton synchronous tracking. First, prediction errors and noise were inevitably contained in the predicted joint angles from the recognition model, by which unsafe and non-smooth motions can be caused when directly applied. The bilateral filter and the hyperbolic tangent-based velocity constraint were consequently integrated to ensure smooth and safe motion. Second, cumulative position drift can be caused by the velocity-based control over time. The position-velocity hybrid strategy with error feedback was adopted to correct this drift in real time, maintaining accurate trajectory tracking during prolonged operation.

Specifically, the lower limb joint angles were predicted and subsequently provided to the controller of the lower limb exoskeleton (LLE) with a period of 0.02 s. The exoskeleton was then controlled with a period of 0.1 s. The lower limb joint velocity can be calculated as follows:


$$\begin{array}{l} p_{i}=\frac{1}{5}\overset{5}{\underset{j=1}{\sum }}p_{i}^{j},v_{i}=\frac{\left(p_{i}-p_{i-1}\right)}{0.1}, \end{array}$$ (12)

where $p_{i}^{j}$ represents the *j*-th data received during the *i*-th control period, *p*_*i*_ denotes the mean angle, and *v*_*i*_ is the velocity.

Moreover, the bilateral filter was employed to smooth estimates and to preserve important features. The bilateral filter is defined by a weight coefficient *W*_*i, j*_, which is calculated as :


$$\begin{array}{l} w_{i,j}=s_{i,j}\cdot r_{i,j}, \end{array}$$ (13)

where


$$\begin{array}{l} s_{i,j}=exp\left(-\frac{|j-i{|}^{2}}{2\sigma_{s}^{2}}\right), \end{array}$$ (14)

$$\begin{array}{l} r_{i,j}=exp\left(-\frac{|v_{j}-v_{i}{|}^{2}}{2\sigma_{r}^{2}}\right). \end{array}$$ (15)

In Equation 13, *s*_*i, j*_ and *r*_*i, j*_ represent the distance and velocity similarity between the *v*_*i*_ and *v*_*j*_, respectively. In Equations 14 and 15, σ_*s*_ and σ_*r*_ are the standard deviation for the distance and velocity similarity, respectively.

A differentiable formulation based on the hyperbolic tangent function was employed to restrict the exoskeleton joint velocities. In contrast to hard saturation, this differentiable formulation avoids discontinuities in the acceleration profile, thereby reducing mechanical jerk and ensuring compliant interaction at the safe boundaries. The formulation is defined as:


$$\begin{array}{l} v=\left\{ \begin{array}{l} \sigma_{1}+\sigma_{2}\times \text{tanh}\left(\frac{v-\sigma_{1}}{\sigma_{2}}\right)\;v>\sigma_{1}\\ v\;-\sigma_{1}\leq v\leq \sigma_{1}\\ -\sigma_{1}+\sigma_{2}\times \text{tanh}\left(\frac{v+\sigma_{1}}{\sigma_{2}}\right)\;v<-\sigma_{1}, \end{array}\right. \end{array}$$ (16)

where σ_1_ and σ_2_ denote the maximum velocity constraints of the joint. For velocities within the interval [−σ_1_, σ_1_], the given velocity can be implemented without modification. For velocities falling outside, the hyperbolic tangent smoothly compresses the magnitude into the prescribed range, thereby preventing the joint from exceeding allowable velocities.

Finally, a position-velocity hybrid control strategy based on error feedback was adopted to mitigate cumulative deviations. Joint velocities were used as the primary motor control input to enable rapid response during exoskeleton motion. However, deviations from the trajectories predicted by the recognition model were inevitably observed in the joint angles over time. To compensate for the drift, the position control component was integrated into the framework, through which the joint angles were rapidly corrected and aligned with the desired motion trajectory.

## 5 Experiments and discussion

### 5.1 Experiment platform and data acquisition

**Figure 3:**
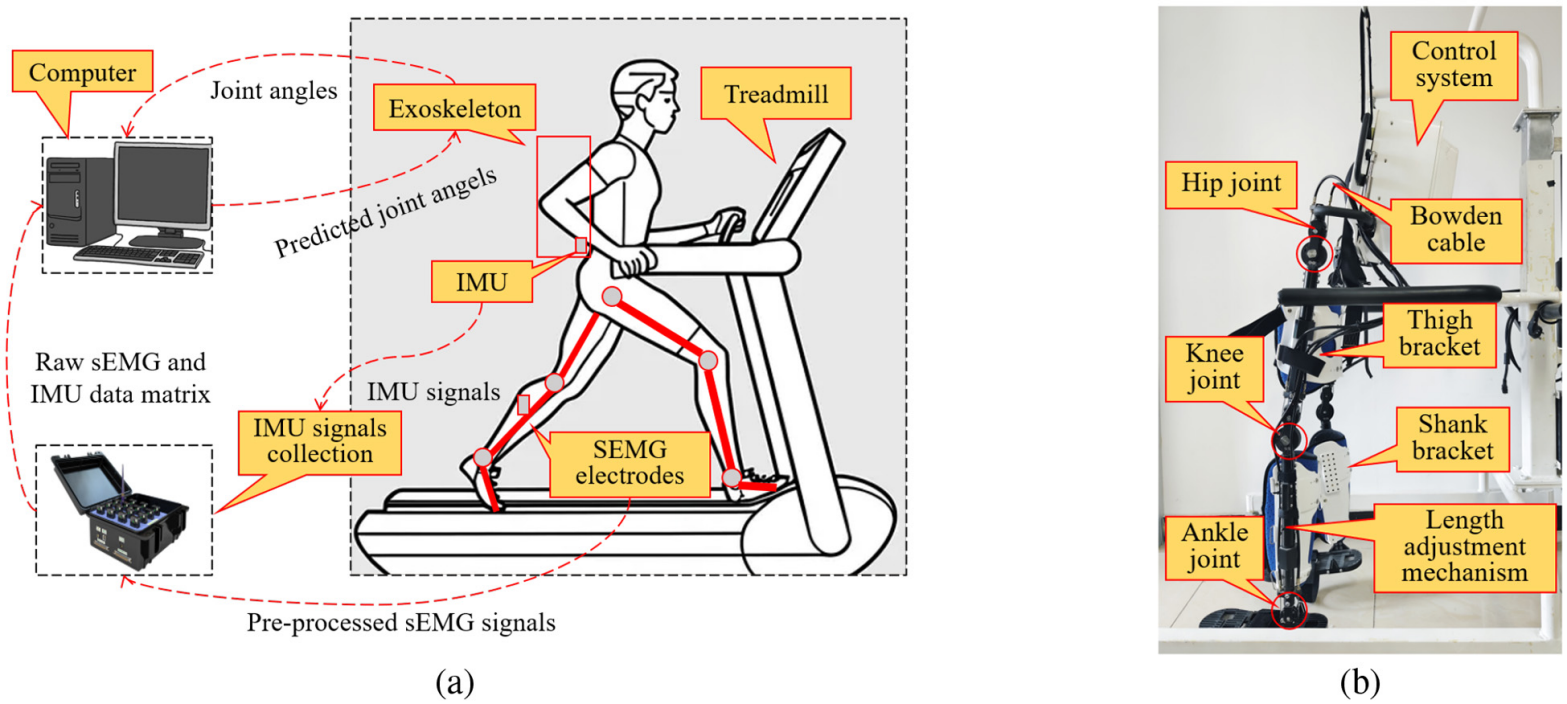
Active physical human–exoskeleton interaction experiment platform. **(a)** Experiment setup for treadmill walking with an exoskeleton and predicted joint angles. sEMG electrodes and IMU were attached to the subject's limbs to record muscle activity and motion signals. These signals are collected by the sEMG and IMU acquisition system and transmitted to the computer for processing. Then, the joint angles were predicted by the computer and sent to the exoskeleton while the subject walks on the treadmill. **(b)** The LLE developed in our institute.

As shown in Figure 3, the LLE developed at the Institute of Automation, Chinese Academy of Sciences, was used as the experiment platform. The LLE has two biomimetic legs, each of which consists of three joints, namely the hip, knee and ankle joints. The Bowden cable was used to transmit the motor force to drive the LLE. The shank bracket and thigh bracket were designed to support more comfortable wear. In addition, the control system, including motors, controller and sensors, was mounted on the backpack. The methods proposed in this study can be applied in the LLE for active rehabilitation training.

**Figure 4:**
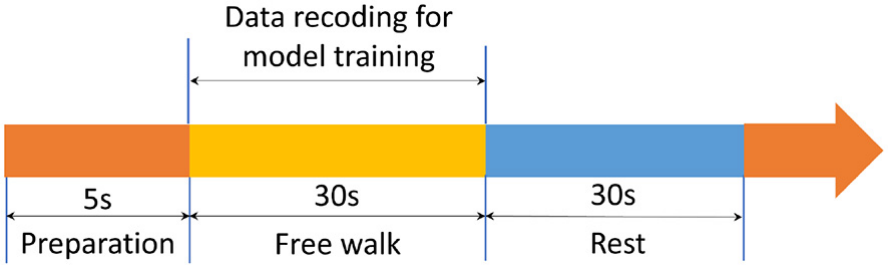
Experiment paradigm for data collection.

Five subjects in our institution participated in the experiment. The offline and online experiments were carried out for each subject, and the validation experiments were also conducted. Each subject was required to perform eight trials for data collection, and each trial was executed in accordance with the experiment paradigm, as shown in Figure 4. In each trial, the subject was first allowed a 5-second preparation period, followed by 30 s of free walking, and then a 30-s rest period.

### 5.2 Intention recognition model results

The proposed recognition model was implemented using PyTorch. All experiments were performed on an NVIDIA RTX A6000 with 48 GB of memory. The Adam optimizer was employed, and the optimization hyperparameters are summarized in Table 2. The batch size was 256 during the model training. For the proper evaluation of the proposed methods, 5-fold cross-validation was employed in the following offline experiments. To prevent data leakage, the training and test datasets were partitioned at the trial level. Specifically, data from different trials were assigned to either the training set or the test set, ensuring no temporal overlap was existed between them.

**Table 2:** Training configuration and optimization hyperparameters of the proposed model.

Hyperparameter	Value
Optimizer	Adam
Loss function	L1 loss (MAE)
Initial learning rate	1 × 10^−3^
Batch size	256
Number of epochs	1000

Three offline experiments were conducted to validate the proposed hybrid neural network. In the first experiment, the prediction accuracy of the proposed model was validated by the datasets recorded from five subjects. The mean square error (MSE), the mean absolute error (MAE), MAPE and the R-square (*R*^2^) were calculated. The results, presented in Table 3, indicate that the hip, knee, and ankle joint angles can be accurately predicted by the proposed method. It was also observed that the ankle predictions exhibit lower accuracy compared the hip and knee. This may be attributed to the limited number of muscles selected and the reduced information content inherent in the sEMG signals associated with ankle motion.

**Table 3:** Prediction errors of the hybrid neural network (subject-equal statistics).

Evaluation metric	Joints	S1	S2	S3	S4	S5	Mean ± Std
MSE (°)	Hip	0.67	0.66	0.47	0.80	1.07	0.73 ± 0.22
	Knee	1.19	1.61	0.88	1.71	3.43	1.76 ± 0.99
	Ankle	2.89	4.09	4.04	2.10	3.40	3.30 ± 0.84
MAE (°)	Hip	0.55	0.57	0.41	0.62	0.64	0.56 ± 0.09
	Knee	0.71	0.87	0.64	0.90	1.21	0.87 ± 0.22
	Ankle	0.82	1.15	0.98	0.77	1.67	1.08 ± 0.36
MAPE (%)	Hip	4.57	5.57	6.48	4.74	6.34	5.54 ± 0.80
	Knee	2.14	4.44	5.80	3.00	4.42	3.96 ± 1.28
	Ankle	1.26	1.69	1.55	1.11	2.29	1.58 ± 0.46
*R* ^2^	Hip	1.00	1.00	0.99	1.00	0.97	0.99 ± 0.01
	Knee	0.99	0.99	0.99	0.99	0.99	0.99 ± 0.00
	Ankle	0.99	0.96	0.98	0.99	0.97	0.98 ± 0.01

Two ablation experiments were conducted. The first ablation experiment was carried out to validate the contribution of different signal combinations. Specifically, CoG data and joint angles were integrated with sEMG signals in various configurations, and the effects on prediction performance were evaluated, as summarized in Table 4. The results demonstrate that the complete fusion of sEMG, angle, and CoG data achieved the best performance across all joints. When CoG data was removed (SEMG + Angle), the hip joint MAPE increased from 5.54% to 8.41%, representing a 51.8% degradation. Similarly, removing joint angle data (SEMG + CoG) resulted in even more substantial performance decline, with hip joint MAPE increasing to 7.14%. Using sEMG signals alone yielded the poorest results, with hip joint MAPE reaching 7.52%. These findings confirm that the synergistic integration of these heterogeneous signals enables more accurate and robust joint angle prediction. This can be attributed to the complementary information provided by the three modalities. Specifically, movement intention is recognized from sEMG signals through the muscle activation patterns that precede actual movement, kinematic continuity is provided by joint angles, and whole-body balance dynamics are reflected by CoG data.

**Table 4:** The ablation experiment results using different input signal combinations.

Input signals	Joint	MSE (°)	MAE (°)	MAPE (%)	*R* ^2^
SEMG + Angle + CoG	Hip	0.73	0.56	5.54	0.99
	Knee	1.76	0.87	3.96	0.99
	Ankle	3.30	1.08	1.58	0.98
SEMG + Angle	Hip	1.89	1.06	8.41	0.98
	Knee	4.82	1.72	7.96	0.99
	Ankle	5.25	1.68	2.37	0.96
SEMG + CoG	Hip	5.52	1.81	7.14	0.98
	Knee	2.26	1.31	3.81	0.99
	Ankle	3.60	1.38	1.95	0.98
CoG + Angle	Hip	1.83	1.62	8.91	0.97
	Knee	4.14	1.79	4.38	0.98
	Ankle	4.01	1.93	4.41	0.98
SEMG	Hip	5.80	1.97	7.52	0.97
	Knee	3.26	1.55	4.57	0.99
	Ankle	4.81	1.81	3.38	0.98

The second ablation experiment was conducted to evaluate the contribution of each architectural component. Three variants were compared against the proposed model: (1) The Mamba fusion module was removed, and the concatenated CNN features were directly fed into the MLP, namely 1D CNN + MLP; (2) The 1D CNN feature extractor was replaced with handcrafted time-domain features namely Handcrafted + Mamba + MLP; (3) The MLP output module was replaced with a single fully connected layer while the 1D-CNN and Mamba were kept unchanged, namely CNN + Mamba + FC. All variants were trained and evaluated under the same experimental settings, and the results are summarized in Table 5. The best overall performance was achieved in the proposed architecture, confirming the effectiveness of each component in the hybrid network.

**Table 5:** The results of architecture ablation experiments.

Inputs		MSE (°)	MAE (°)	MAPE (%)	*R* ^2^
1DCNN + Mamba + MLP	Hip	0.73	0.56	5.54	0.99
	Knee	1.76	0.87	3.96	0.99
	Ankle	3.30	1.08	1.58	0.98
1DCNN + MLP	Hip	0.65	0.54	10.15	0.99
	Knee	1.74	0.87	10.64	0.98
	Ankle	2.41	0.88	1.26	0.98
Handcrafted + Mamba + MLP	Hip	0.50	0.47	9.13	0.99
	Knee	1.30	0.77	11.69	0.98
	Ankle	5.00	1.25	1.81	0.97
1DCNN + Mamba+ FC	Hip	0.82	0.63	15.00	0.99
	Knee	2.67	1.13	16.31	0.99
	Ankle	9.20	1.75	2.64	0.96

For further evaluation, a comparative experiment was carried out using several representative methods in the literature, including long short-term memory (LSTM) networks (Song et al., 2023), random forest with principal component analysis (RFPCA) (Li et al., 2020c), CNN-LSTM (Jiao et al., 2021) and support vector regression (SVR). The comparative results are given in Table 6. Considering all performance metrics, the proposed approach demonstrates consistently lower prediction errors and higher coefficients of determination (*R*^2^), thereby validating its effectiveness and robustness for joint angle prediction.

**Table 6:** The comparative experiment results using different methods.

Inputs		MSE (°)	MAE (°)	MAPE (%)	*R* ^2^
Ours	Hip	0.73	0.56	5.54	0.99
	Knee	1.76	0.87	3.96	0.99
	Ankle	3.30	1.08	1.58	0.98
LSTM	Hip	1.41	1.61	5.00	86.54
	Knee	1.97	2.65	1.92	85.33
	Ankle	1.62	1.83	3.12	81.61
RFPCA	Hip	4.71	4.69	1.14	67.53
	Knee	4.87	4.73	2.48	73.32
	Ankle	5.13	5.35	1.14	67.52
CNN-LSTM	Hip	5.28	4.15	4.23	7.34
	Knee	4.22	3.25	4.87	16.25
	Ankle	4.36	5.01	3.29	4.35
SVR	Hip	7.31	6.38	3.51	4.01
	Knee	6.12	3.54	4.85	3.15
	Ankle	5.72	4.19	4.37	10.55

### 5.3 Validation of online adaptive strategy

The validation experiment for the proposed online adaptive strategy was conducted through two pseudo-online experiments, in which the discrete Fréchet distance (FD) was used to quantify the similarity between the joint angle trajectories of different methods. The FD is defined as:


$$\begin{array}{l} \xi (s,\text{l})=\underset{\sigma }{\text{min}}\;\underset{(i,j)\in \sigma }{\text{max}}||s_{i}-l_{j}|| \end{array}$$ (17)

where **s** = {*s*_1_, ..., *s*_*n*_} and **l** = {*l*_1_, ..., *l*_*m*_} denote the trajectories, and σ is the index pairs from (1, 1) to (*n, m*)

Firstly, the sEMG signals, the CoG data and the historical joint angles used for prediction were recorded simultaneously during a 6-min trial. The recorded data were subsequently utilized to predict the lower joint angles with and without the proposed PCA-STM method. Following the initial data collection, the joint angles were continuously predicted using the trained models. To provide a detailed temporal analysis, the 6-min trial was evenly divided into six consecutive segments, denoted as *t*_1_, *t*_2_, …, *t*_6_. Moreover, a fine-tuning strategy was also implemented as a baseline adaptive method. In this approach, the convolutional and Mamba layers of the pre-trained model were frozen, and only the MLP layers were fine-tuned using the incrementally accumulated calibration data. For each segment, the model was fine-tuned for 20 epochs with a learning rate of 1 × 10^−4^ using the Adam optimizer, and the calibration pool was expanded by appending the data from the completed segment. The MAPE values of the hip, knee, and ankle joint angles were calculated for each segment, as presented in Table 7.

**Table 7:** Results of the pseudo online experiments for adaptive strategy.

Method	Joints	t1	t2	t3	t4	t5	t6	Mean ± Std
Normal	Hip	11.04	12.19	13.61	11.07	16.88	12.49	12.88 ± 1.99
	Knee	2.36	2.49	1.89	2.53	1.83	2.72	2.30 ± 0.33
	Ankle	1.76	3.68	2.25	2.76	2.66	2.53	2.61 ± 0.58
PCA-STM	Hip	9.21	9.94	8.72	8.60	7.32	7.09	8.48 ± 1.00
	Knee	1.83	2.29	1.85	1.96	1.59	2.17	1.95 ± 0.23
	Ankle	1.09	2.39	1.35	1.74	1.45	1.62	1.61 ± 0.41
Fine-tuning	Hip	10.97	12.80	13.86	11.48	13.84	9.63	12.10 ± 1.55
	Knee	2.15	2.19	2.88	2.25	2.85	2.53	2.48 ± 0.30
	Ankle	1.32	2.91	1.58	2.01	1.77	1.93	1.92 ± 0.50

As shown in Table 7, the lowest mean MAPE values across all three joints were achieved by the PCA-STM method. Moreover, a consistent decreasing trend in the hip MAPE was observed for PCA-STM across the six segments (from 9.21% in *t*_1_ to 7.09% in *t*_6_), indicating that the adaptive capability was progressively enhanced over time. The smallest standard deviations were also obtained by PCA-STM for all joints, suggesting that more stable predictions were maintained throughout the trial. In contrast, the fine-tuning method was found to yield only marginal improvements over the Normal baseline for the hip joint, while a slightly higher MAPE was observed for the knee joint. These results demonstrate that the proposed PCA-STM strategy is more effective in mitigating performance degradation caused by temporal variations in sEMG signals.

**Figure 5:**
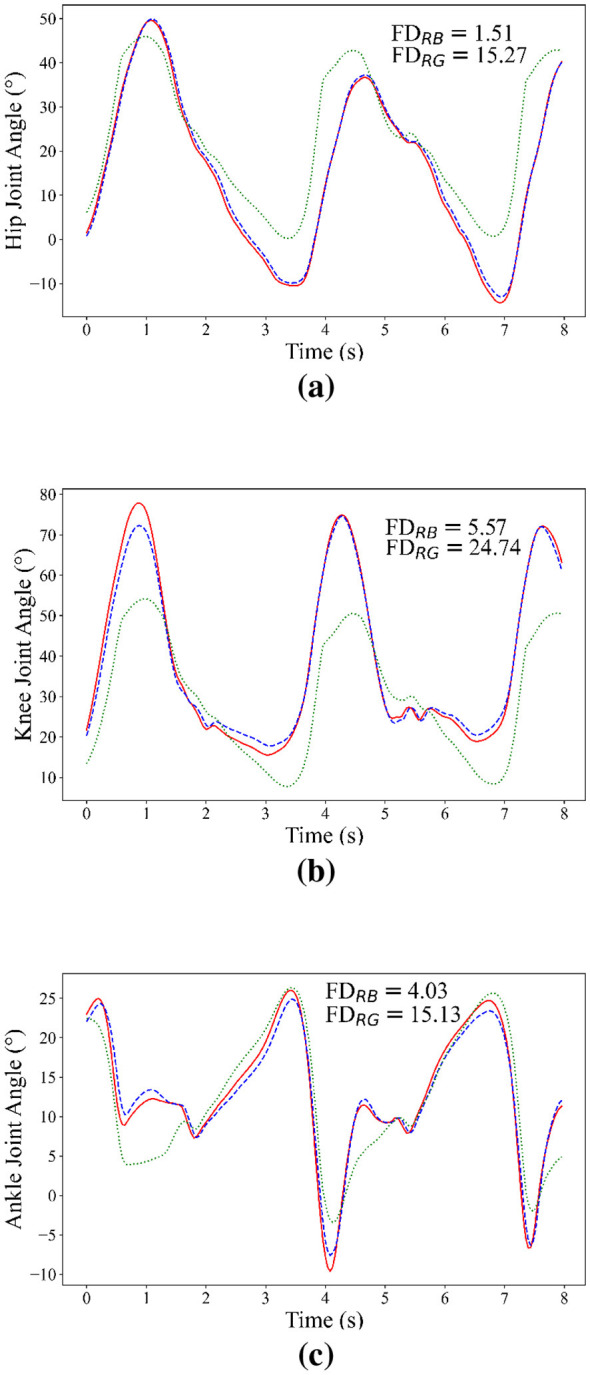
Predicted results in the long-term experiment. The green, blue and red lines represent measured angles, predicted angles from the original model and predicted angles from the adaptive model, respectively. **(a)** Hip joint. **(b)** Knee joint. **(c)** Ankle joint.

The joint angles for one subject over 8 s are given in Figure 5. To further quantify the differences among trajectories, FD values between different curves are also reported. The *FD*_*RB*_ represents the distance between the measured joint angles and the predicted joint angles with the proposed adaptive method. The *FD*_*RG*_ denotes the distance between the measured joint angles and the predicted joint angles without the adaptive method.

The degradation of prediction accuracy was observed in the model without the adaptive method, characterized by an increase in the FD. By contrast, the degradation of prediction accuracy was mitigated by the proposed PCA-STM method, resulting in preserved geometric similarity between the predicted and measured joint trajectories. Based on those results, the effectiveness of the proposed PCA-STM method can be confirmed.

### 5.4 Validation of the control framework

To validate the proposed exoskeleton control framework, a subject from our institute walked on a treadmill at a self-selected speed for 3 min, during which the exoskeleton was driven by the predicted angles. In the experiment, the lower-joint angles of the subject were measured, and the corresponding joint angles of the exoskeleton were simultaneously collected and recorded.

Before the experiment, several parameters in Equations 13 and 16 were given. In Equation 16, the sum of σ_1_ and σ_2_ defines the upper and lower bounds of the constraint function. In the experiment, the sum of σ_1_+σ_2_ was given as 90% of the maximum joint velocity of the exoskeleton. The σ_1_ represents the turning point of the constraint function, which was determined by the maximum joint velocity of the subject to reduce excessive joint motion. In Equations 14 and 15, σ_*s*_ and σ_*r*_ were determined by the variances of the spatial domain and the value domain, respectively.

**Figure 6:**
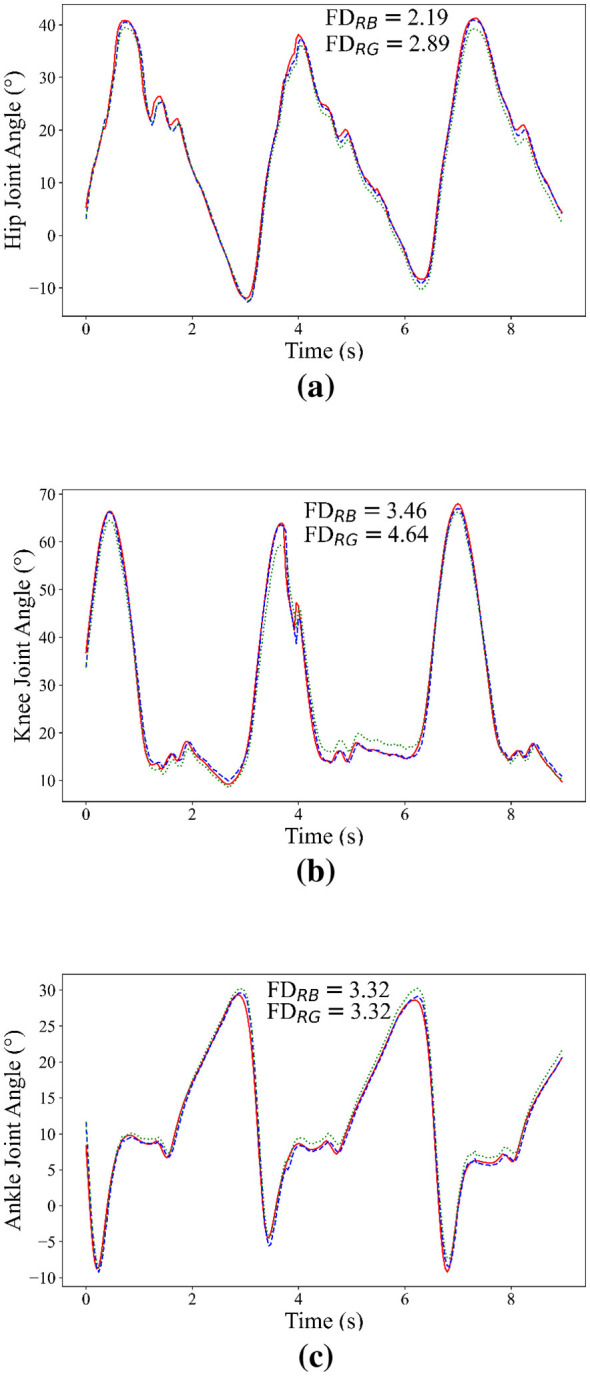
Predicted results in the long-term experiment. The green, blue and red lines represent angles measured from angle sensors, predicted by the proposed model, and recorded by the exoskeleton, respectively. **(a)** Hip joint. **(b)** Knee joint. **(c)** Ankle joint.

The results of the experiment were presented in Figure 6. The FDs were also calculated to quantify the similarity between different trajectories. As shown in the Figure 6, it can be seen that the lower limb angles of the subject were synchronously tracked by the exoskeleton. Nevertheless, discrepancies between the subject joint angles and the exoskeleton joint angles were observed, which may be attributed to suboptimal parameter settings within the control framework.

## 6 Conclusion and future work

In this study, an integrated framework for active physical human-exoskeleton interaction was developed, where motion intention recognition, adaptive modeling, and synchronous tracking were focused on. First, a multimodal joint angle prediction model was constructed by combining sEMG signals, CoG data, and historical joint angles through a hybrid neural network composed of 1D-CNN, Mamba, and MLP algorithms. Second, to mitigate the performance degradation caused by the non-stationary characteristics of sEMG signals, an online adaptive algorithm based on PCA-STM was employed, by which the joint angle prediction accuracy and the adaptivity of the designed model were improved efficiently. Third, a hierarchical control strategy was designed to realize synchronized motion between the human and the exoskeleton, and the position-velocity hybrid controller with bilateral filtering and non-linear velocity constraints was used to realize the synchronous tracking based on the predicted joint angles. In future work, the proposed method will be evaluated in practical scenarios to validate its feasibility in real-world environments.

## Data Availability

The raw data supporting the conclusions of this article will be made available by the authors, without undue reservation.
